# Salidroside Supplementation Affects In Vitro Maturation and Preimplantation Embryonic Development by Promoting Meiotic Resumption

**DOI:** 10.3390/genes14091729

**Published:** 2023-08-30

**Authors:** Shuming Shi, Zhaojun Geng, Xianfeng Yu, Bing Hu, Liying Liu, Zhichao Chi, Linyi Qu, Mingjun Zhang, Yongxun Jin

**Affiliations:** 1 Jilin Provincial Key Laboratory of Animal Model, College of Animal Science, Jilin University, Changchun 130062, China; shism20@mails.jlu.edu.cn (S.S.); gengzj20@mails.jlu.edu.cn (Z.G.); xianfeng79@jlu.edu.cn (X.Y.); liuliying22@mails.jlu.edu.cn (L.L.); chizc9919@mails.jlu.edu.cn (Z.C.); quly9919@mails.jlu.edu.cn (L.Q.); 2 Animal Genome Editing Technology Innovation Center, College of Animal Science, Jilin University, Changchun 130062, China; hubing21@mails.jlu.edu.cn

**Keywords:** embryos, MAPK, maturation, mitochondria, oocytes, Sal, ROS

## Abstract

**Simple Summary:**

The in vitro maturation (IVM) of oocytes is often delayed due to oxidative stress and mitochondrial function, resulting in a decrease in oocyte quality. We used a porcine oocyte model to check the effects of salidroside supplementation during IVM on the quality and developmental competence of oocytes. Using a variety of experimental methods, the efficiency of salidroside in protecting mature oocytes, apoptosis, and mitochondrial function was observed.

**Abstract:**

Salidroside (Sal) possesses several pharmacological activities, such as antiaging, and anti-inflammatory, antioxidant, anticancer activities, and proliferation-promoting activities, but the effects of Sal on oocytes have rarely been reported. In the present study, we evaluated the beneficial effects of Sal, which is mainly found in the roots of Rhodiola. Porcine cumulus oocyte complexes were cultured in IVM medium supplemented (with 250 μmol/L) with Sal or not supplemented with Sal. The maturation rate in the Sal group increased from 88.34 ± 4.32% to 94.12 ± 2.29%, and the blastocyst rate in the Sal group increased from 30.35 ± 3.20% to 52.14 ± 7.32% compared with that in the control group. The experimental groups showed significant improvements in the cumulus expansion area. Sal reduced oocyte levels of reactive oxygen species (ROS) and enhanced intracellular GSH levels. Sal supplementation enhanced the mitochondrial membrane potential (MMP), ATP level, and mtDNA copy number, which shows that Sal enhances the cytoplasmic maturation of oocytes. Oocytes in the Sal group exhibited slowed apoptosis and reduced DNA breakage. Cell cycle signals and oocyte meiosis play important roles in oocyte maturation. The mRNA expressions of the MAPK pathway and MAPK phosphorylation increased significantly in the Sal group. The mRNA expression of the oocyte meiosis gene also increased significantly. These results show that Sal enhances the nuclear maturation of oocytes. Moreover, Sal increased the number of blastocyst cells, the proliferation of blastocysts, and the expressions of pluripotency genes. Sal down-regulated apoptosis-related genes and the apoptotic cell rate of blastocysts. In summary, our results demonstrate that Sal is helpful to improving the quality of porcine oocytes in vitro, and their subsequent embryonic development.

## 1. Introduction

In vitro porcine embryo production (IVP) is now being used in the development of gamete/embryo biology and agriculture, as well as in the production of cloned and transgenic pigs. Therefore, it has certain advantages for research and commercial use [1,2]. Even though the porcine in vitro maturation system has been established, the maturation rate is not ideal. Due to its sensitivity to the in vitro maturation environment, the quality of oocytes plays a decisive role in embryo quality, and it is necessary to further improve the maturation rate [3,4], lay a foundation for the improvement of human embryo reproduction technology, and strengthen its application in agriculture and medical research [5].

In vitro maturation (IVM) is a complex process controlled by multiple intrinsic and extrinsic factors, studies in mice have shown that perturbations of mitochondrial function affect the maturation of oocytes [6]. Oxidative stress has become a damaging factor of oocytes [7]. Currently, to promote the production of in vitro embryos, supplements are used during the in vitro maturation of oocytes such as antioxidants, which can counteract some of the negative effects of oxidative stress [8]. Mitogen-activated protein kinases (MAPKs) are serine/threonine protein kinases that widely exist in mammalian cells [9]. The MAPK signaling pathway plays an important role in cell proliferation, differentiation, cell survival, and apoptosis [10]. During the process of oocyte maturation, MAPK maintains the stability of the spindle structure and promotes oocyte maturation [5].

Sal is mainly found in the roots of Rhodiola. It has been extensively used for medicinal purposes, because it has many biological and pharmacological properties, and has many active properties [11], such as anticancer [12], antioxidant [13], antiaging, antidiabetic, antidepression, antilipid, anti-inflammatory immune regulation [14], promoting multiplication, and so on [15]. Sal influences the PI3K/Akt, AMPK and MEK/ERK pathways, which are the main reasons for its promotion of proliferation [15,16]. Sal has well-documented beneficial biological and physiological functions such as anticancer, anti-oxidative, and slowing down apoptosis. However, there is little information available in the literature on the mode of action of Sal during oocyte IVM.

Therefore, we hypothesized that Sal would enhance the developmental potential of oocytes because it has a protective effect. More importantly, the molecular mechanisms by which Sal affect oocyte maturation are still unknown. In this study, we used pigs as a model for evaluating whether adding Sal to the culture medium can improve IVM oocyte quality and subsequent embryonic development. We first examined the first polar body excretion and parthenogenetic activation of embryonic development, and then assessed oocytes and preimplantation embryos’ molecular changes, factors affecting cytoplasmic maturation including ROS levels, mitochondrial membrane potential (MMP), and mtDNA copy number, factors affecting nuclear maturation including proliferation and apoptosis levels, and MAPK level changes.

## 2. Materials and Methods

All reagents except those specially marked were purchased from Sigma Aldrich (St. Louis, MO, USA).

### 2.1. Oocyte Collection and Culture

Porcine ovaries were collected from the local slaughterhouse, and then transported to the laboratory in 37 °C 0.9% saline in a thermos. Follicular fluid was extracted from these 3–6 mm follicles using a syringe with a 1.2 mm needle, and collected under a stereomicroscope (SMZ800N, Nikon, Tokyo, Japan). This experiment used cumulus oocyte complexes (COCs), which are oocytes with three or more layers of cumulus cells that are evenly distributed on the surface. The subsequent COCs were washed three times in medium 199 containing HEPES (GIBCO, Waltham, MA, USA, 12350039), 0.1% PVA, and cultured in IVM (tissue culture medium 199 supplemented with 0.6 mM L-cysteine, 0.91 mM Na pyruvate, 10 ng/mL EGF, 10% porcine follicular fluid, 10 IU/mL follicle-stimulating hormone, 10 IU/mL luteinizing hormone, and 0.1% penicillin streptomycin), and in order to explore the optimal dosing concentration of the treatment group, 50 oocytes were placed in 500 μL of IVM supplemented with 50 μM, 250 μM, and 400 μM Sal. They were equally divided into treated and untreated groups, and were added to the treatment group of 250 μM Sal covered with mineral oil incubate in 38.5 °C under 5% CO_2_ and 100% moisture for 42 h. A picture for each COC was taken before and at the end of the in vitro culture, and the cumulus expansion was calculated as the ratio between the final cumulus area and the initial cumulus area. The cumulus area was measured by analysis via NIH ImageJ software 1.52a (National Institutes of Health, Bethesda, MD, USA).

### 2.2. Parthenogenetic Activation

The cumulus cells were removed with 0.1% hyaluronidase, and the oocytes without cumulus cells were treated in 5 mm ionomycin (pzm-5 configuration) for 5 min, then washed in pzm-5 three times. Then, the embryos were placed in bicarbonate-buffered porcine zygote medium 5 (PZM-5) containing 0.4 mg/mL bovine serum albumin (BSA) and 7.5 μg/mL CB for 4 h to inhibit the excretion of the pseudo-second polar body. The blastocyst rates and diameters were analyzed on day 6, and the blastocyst diameters were analyzed via NIH ImageJ software 1.52a (National Institutes of Health, Bethesda, MD, USA).

### 2.3. Count the Number of Cells in the Blastocyst

To quantify the total number of cells in the blastocysts, the blastocysts of the treatment group and untreated group on the seventh day were selected and fixed with 4% paraformaldehyde with pH = 7.4. Then, the fixed blastocysts were washed with PBS–PVA (0.1% PVA) three times, and placed into the permeabilization solution containing 0.5% TritonX-100 for 20 min at room temperature. The blastocysts were again washed with PBS–PVA three times; the 5 ng/mL Hoechst 33,342 stained nuclei were incubated at room temperature for 10 min, then gently placed on slides and sealed with an anti-fluorescence quencher. Next, the blastocysts were gently mounted onto glass slides and examined under a fluorescence microscope (IX73, Olympus, Tokyo, Japan). NIH ImageJ software was used to analyze the total number of nuclei.

### 2.4. Evaluation of ROS and GSH Levels in Oocytes

To measure the ROS and GSH levels in the M2 oocytes of the treatment group and untreated group, the M2 oocytes in each group were divided into four groups and cultured in PBS–BSA medium containing 10 mM 20, 70-dichlorodihydrofluorescein diacetate (Thermo Fisher Scientific, Waltham, MA, USA, C400), and 10 mM 4-chloromethyl-6,8-difluoro-7-hydroxycoumarin (Thermo Fisher Scientific, C12881) for 15 and 30 min. Then, the oocytes were washed with 0.1% PBS–BSA for 3 times. A digital camera (E179168; Nikon) was connected to a fluorescence microscope to take pictures, with the fluorescence photos saved as TIFF files, and the fluorescence intensity was analyzed using NIH ImageJ software.

### 2.5. Mitochondrial Membrane Potential Assay

To measure the mitochondrial membrane potential (MMP), M2-stage oocytes were washed three times with PBS–PVA, and then washed with 2 mM 5,50,6,60-tetrachloro-1,10,3,30-tetraethyl-imidacarbocyanine iodide (JC-1, Beyotime, Haimen, China) for 30 min. The oocytes were again washed three times with PBS–PVA, and a fluorescence microscope was used to connect to the camera and capture the red/green fluorescence signal. The MMP was calculated as the ratio of red fluorescence and analyzed according to the manufacturer’s instructions. The fluorescence intensity of the images was processed and measured using NIH ImageJ software.

### 2.6. Determination of ATP Content

The oocyte ATP content was determined using an Enhanced ATP Assay Kit (Beyotime, China) according to the manufacturer’s instructions, and the results are shown in the microplate reader.

### 2.7. Mitochondrial Copy Number Detection

The total DNA was extracted from 200 MII-stage oocytes using a DNA isolation kit (QIAGEN, Hilden, Germany, 80284) according to the instructions. Through fluorescence quantification to detect the mitochondrial copy number, its principle is to extract mitochondrial *ATP6*, *ND1*, and *COX1* genes, and the nuclear single-copy gene *GCG* was used to detect the relative concentrations of the mitochondrial and nuclear genomes. Since the genes in mitochondria are single-copy genes, the mitochondrial gene and single-copy nuclear gene relative concentration ratio can be used to determine a single tissue cell. Quantitative fluorescence PCR was performed using the total DNA as the template, and each sample was quantified using specific primers for mitochondrial genes and nuclear single-copy genes. The statistics using the formula for the PCR primers used to amplify each gene are shown in Table 1.

For the quantification of the mitochondrial DNA copy number, using the single-copy nuclear gene *GCG* as the reference gene, the multiple of the mitochondrial gene expression relative to *GCG* is the mitochondrial copy number of cells in the tissue. The calculation formula is as follows:

N = 2^−Ctmt^/2^−Ctn^

N: mtDNA copy number; Ctmt: Ct value for mitochondrial genes; Ctn: Ct value for nuclear single-copy genes.

### 2.8. Quantitative Real-Time PCR (qRT-PCR)

The total mRNA was extracted from 200 M2 stage oocytes and 50 blastocysts using an mRNA isolation kit (QIAGEN, Germany, 80284) according to the manufacturer’s instructions. The gene expressions were quantified using the CFX96 real-time system (Bio-Rad, Harkles, CA, USA). The internal control group was the expression level of GAPDH. The statistics for the formula for the PCR primers used to amplify each gene are shown in Table 2.

### 2.9. Western Blotting

A total of 200 M2 stage oocytes were collected, and the protein was extracted by RIPA (Beyotime, P0013 K); a 10–15% sodium dodecyl sulfate (SDS) separation gel and a concentration gel were prepared. The diluted primary antibodies H_2_AX (1:2000, 10856-1-AP, Proteintech, Rosemont, IL, USA) and p44/42 MAPK (1:1000, 9101, Cell Signaling) were added to the membrane and incubated overnight at 4 °C. The membranes were washed five times with TBST (10 min each time), supplemented with horseradish peroxidase-marked secondary antibody (1:5000; Proteintech, SA00001–1), oscillated, and then incubated at room temperature for 1 h. After incubation, each membrane was washed five times with TBST (10 min each time) and reacted with enhanced chemiluminescence solution (Tanon, 180–5001) at room temperature for 5 s, and the relative expressions of target proteins were measured using GAPDH (1:5000, 10494-1-AP, Proteintech) as the reference.

### 2.10. Assessment of Blastocyst Apoptosis

For the TUNEL (Beyotime, C1086S) assays, the blastocysts were incubated with 50 μL of TUNEL reaction mixture for 1 h at 37 °C in the dark. A fluorescence microscope connected to a digital camera was used to take pictures to capture the fluorescence signal. The images were processed and measured using NIH ImageJ software.

### 2.11. Statistics of Blastocyst Cell Proliferation

The propagation of blastocyst cells was cytochemically detected according to the manufacturer’s instructions (C0071L, Beyotime, China). Briefly, the blastocysts were incubated with the EdU staining buffer for 1 h, fixed with 4% polyformaldehyde, and stained for nuclei with Hoechst. The stained blastocysts were scanned and photographed under a microscope, and the numbers of positive cells were counted.

### 2.12. Statistical Analysis

All of the data were analyzed using SPSS software version 11.0 (IBM, Armonk, NY, USA). All of the experiments were performed more than 3 times. Comparisons of data between groups were performed with Student’s *t*-test. The values are expressed as the mean ± standard deviation, and significant differences are represented with * (*p* < 0.05), ** (*p* < 0.01), and *** (*p* < 0.001).

## 3. Results

### 3.1. Protective Effects of Sal at Different Concentrations on Porcine Oocytes

**Figure 1:**
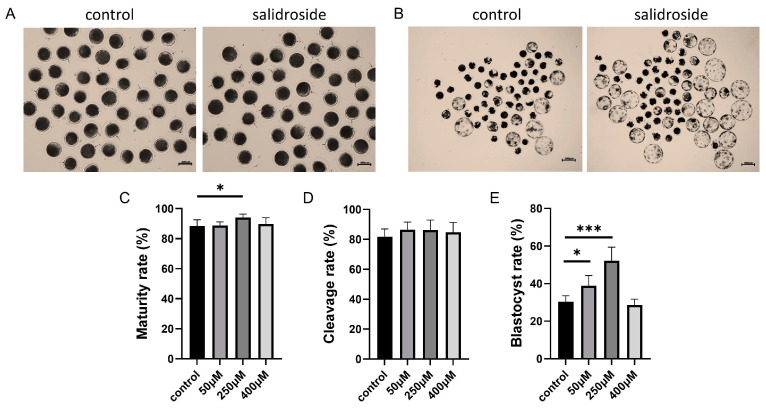
Protective effects of Sal at different concentrations on porcine oocytes. (**A**) Representative images of oocyte PBE in the control and Sal groups. The black arrow indicates an oocyte with the first polar body. Scale bar = 100 μm. (**B**) Representative images of embryo development on day 6 in the control and Sal groups. Scale bar = 200 μm. (**C**) Maturation rate of oocytes in the control (n = 292) and Sal groups (50 μM n = 301; 250 μM n = 296; 400 μM n = 294). (**D**) Cleavage rates of porcine parthenogenetic embryos at 30 h after parthenogenetic activation in the control (n = 281) and Sal groups (50 μM n = 286; 250 μM n = 272; 400 μM n = 278). (**E**) Blastocyst formation rates of porcine parthenogenetic embryos on day 6 in the control (n = 281) and Sal groups (50 μM n = 286; 250 μM n = 272; 400 μM n = 278). The data were obtained from three separate experiments. Values are expressed as the mean ± standard deviation, and significant differences are represented with * (*p* < 0.05) and *** (*p* < 0.001).

To determine the optimal concentration of Sal, different concentrations of Sal (0 μM, 50 μM, 250 μM, 400 μM) were added to the IVM culture medium. After 44 h of culture, the results showed that compared with the control group, Sal had the highest maturity rate at the concentration of 250 μM (Figure 1A,C 88.34 ± 4.32% vs. 88.83 ± 2.36% vs. 94.12 ± 2.29% vs. 89.71 ± 4.25%). The cleavage rate after 30 h showed statistically significant parthenogenetic activation, but there was no significant difference in the cleavage rate (Figure 1D 81.78 ± 5.18% vs. 86.40 ± 5.16% vs. 86.22 ± 6.65% vs. 84.76 ± 6.53%). The blastocyst rate was counted on the sixth day of parthenogenetic activation. The blastocyst rate was also the highest among oocytes under 250 μM Sal culture (Figure 1B,E 30.35 ± 3.20% vs. 38.89 ± 5.38% vs. 52.14 ± 7.32% vs. 28.58 ± 3.21%), and had the best protective effect on oocytes.

**Figure 2:**
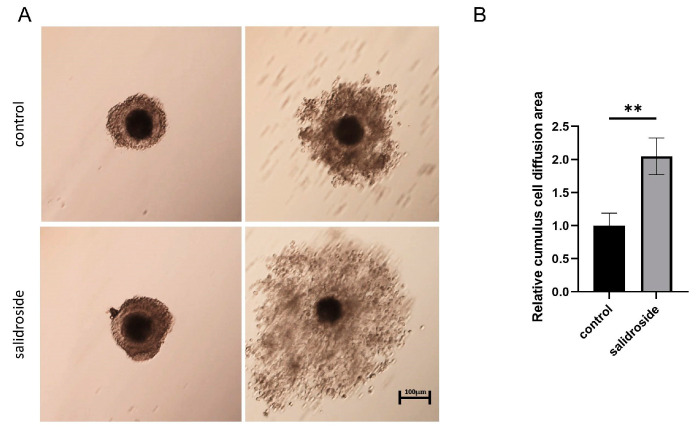
Protective effects of Sal at different concentrations on porcine oocytes. (**A**) Representative images of cumulus cell expansion status before and after IVM in the control and Sal groups. Scale bar = 100 μm. (**B**) Relative cumulus cell expansion areas in the control (n = 31) and Sal groups (n = 33). The data were obtained from three separate experiments. Significant differences are represented with ** (*p* < 0.01).

Based on the above results, a concentration of 250 μM was used in the follow-up experiment. Photographs of each COC were taken before and after in vitro culture, and the ratio between the final cumulus area and the initial cumulus area was calculated. The Sal treatment group significantly increased the area of cumulus expansion (Figure 2A,B). To investigate the reasons for the increase in cumulus expansion area, we collected digested cumulus cells for flow cytometry reactive oxygen species detection and mitochondrial DNA copy number detection, and found that the data in the treatment group were higher than those in the control group (Figure S1).

### 3.2. Effects of Sal Supplementation on the Levels of Oxidative Stress in Porcine Oocytes

**Figure 3:**
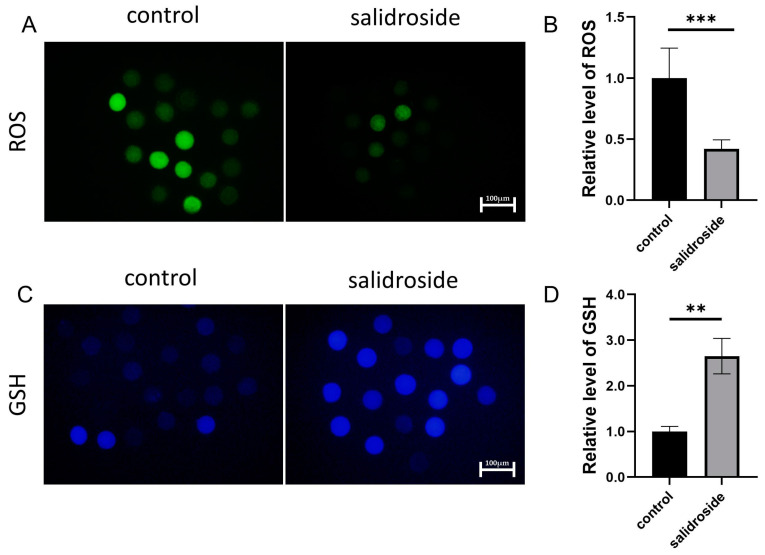
Effects of Sal supplementation on the levels of oxidative stress in porcine oocytes. (**A**) Representative fluorescence images of intracellular H_2_DCFDA (ROS) levels in the control and Sal groups. Scale bar = 100 μm. (**B**) Representative fluorescence images of intracellular glutathione (ROS) levels in the control (n = 62) and Sal groups (n = 59). Scale bar = 100 μm. (**C**) Relative intracellular GSH levels in the control and Sal groups. (**D**) Relative intracellular GSH levels in the control (n = 53) and Sal groups (n = 54). Significant differences are represented with ** (*p* < 0.01) and *** (*p* < 0.001). The data were obtained from three separate experiments.

Since Sal has free radical scavenging properties, we further evaluated whether Sal supplementation during IVM can improve the oxidative stress of porcine oocytes. As shown in Figure 3A,B, the ROS level in the porcine oocytes in the Sal group was significantly lower than that in the control group during the M2 stage, and Sal was not added during IVM. Based on these results, we further evaluated the level of GSH in porcine M2 oocytes, as shown in Figure 3C,D. Compared with the control group, the GSH level in the Sal-supplemented group was significantly higher.

### 3.3. Effects of Sal Supplementation on the Mitochondrial Function of Porcine Oocytes

**Figure 4:**
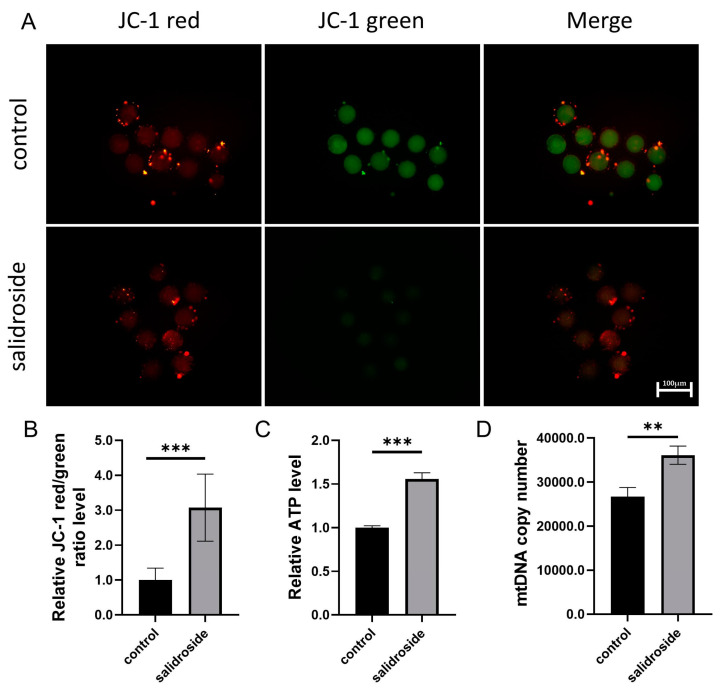
Effects of Sal supplementation on the mitochondrial function of porcine oocytes. (**A**) Representative fluorescence images of JC-1 fluorescence staining in the control and Sal groups. Scale bar = 100 μm. (**B**) Relative intracellular MMP levels in the control (n = 40) and Sal groups (n = 36). (**C**) Relative ATP levels in the control and Sal groups. (**D**) Mitochondrial DNA copy numbers in the control and Sal groups. The data were obtained from three separate experiments. Significant differences are represented with ** (*p* < 0.01) and *** (*p* < 0.001).

Mitochondria are the energy factories of oocytes, one of the indicators of oocyte quality, and play an important role in cell metabolism, growth, and proliferation [17]. We used the JC-1 fluorescence reaction to evaluate the mitochondrial membrane potential (MMP) of the oocytes. Representative images of JC-1 staining are shown in Figure 4A. The quantitative results showed that the average relative value of fluorescence intensity (red/green) in the Sal-supplemented group was significantly higher than that in the control group (Figure 4B). We measured the concentration of ATP in oocytes between different treatment groups, and found that the ATP concentration in the treatment group treatment was significantly higher than that in the control group (Figure 4C). Some studies have suggested that the mitochondrial DNA (mtDNA) copy number could be used as a biomarker of human oocyte viability [18]. We measured that the mtDNA copy number in oocytes between different treatment groups was significantly higher than that in the control group (Figure 4D).

### 3.4. Effects of Sal Supplementation on Apoptosis and DNA Damage in Porcine Oocytes

**Figure 5:**
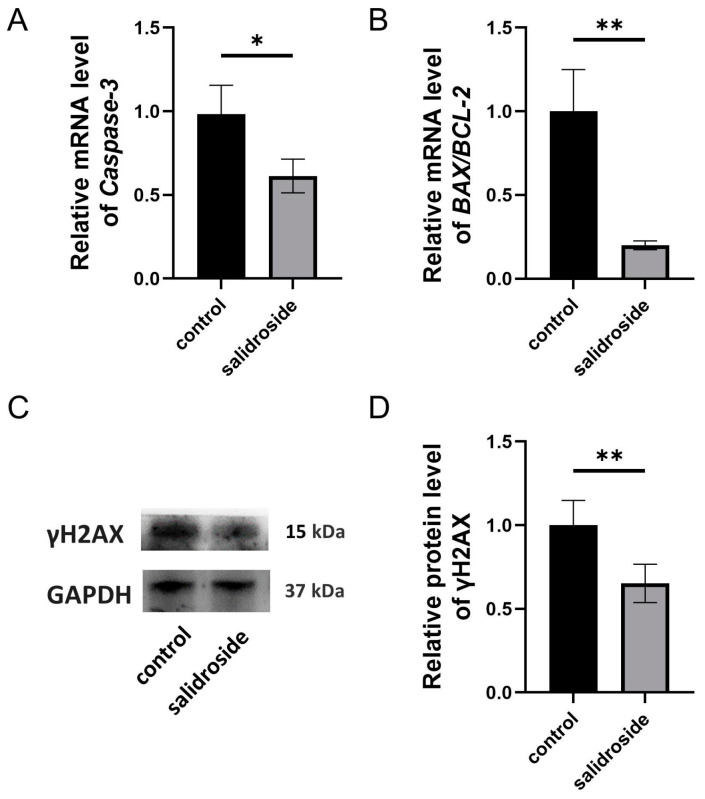
Effects of Sal supplementation on apoptosis and DNA damage in porcine oocytes. (**A**) Relative gene expressions of *Caspase-3* in the control and Sal groups. (**B**) Relative gene expressions of *BAX*/*BCL-2* in the control and Sal groups. (**C**) The protein expression levels of γH2AX in the control and Sal groups. (**D**) Relative levels of γH2AX in the control and Sal groups. The data were obtained from three separate experiments. Significant differences are represented with * (*p* < 0.05), and ** (*p* < 0.01).

Based on the mRNA expression, Sal significantly down-regulated the apoptosis related gene *Caspase-3* (Figure 5A) and the mRNA expression of *BAX*/*BCL-2* (Figure 5B), and significantly down-regulated the DNA breakage marker protein γH2AX (Figure 5C,D).

### 3.5. Effects of Sal Supplementation on MAPK Activation and Meiosis in Porcine Oocytes

**Figure 6:**
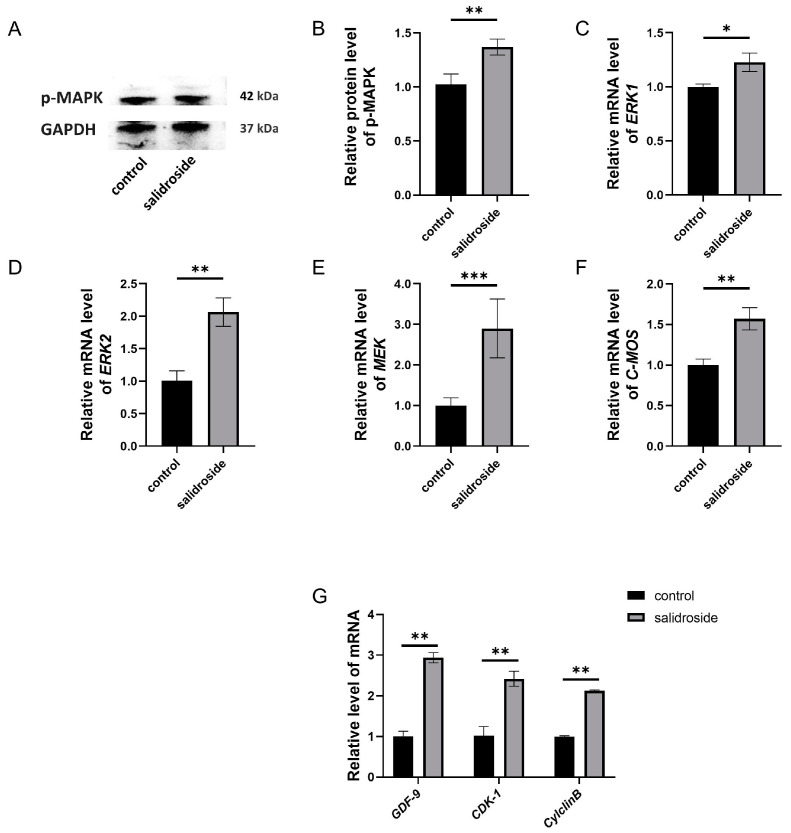
Effects of Sal supplementation on MAPK activation and meiosis of porcine oocytes. (**A**) The protein expression levels of p-MAPK in the control and Sal groups. (**B**) Relative levels of p-MAPK in the control and Sal groups. (**C**) Relative gene expressions of *ERK1* in the control and Sal groups. (**D**) Relative gene expressions of *ERK2* in the control and Sal groups. (**E**) Relative gene expressions of *MEK* in the control and Sal groups. (**F**) Relative gene expressions of *C-MOS* in the control and Sal groups. (**G**) Relative gene expressions of *GDF-9*, *CDK-1*, and *Cyclin B* in the control and Sal groups. The data were obtained from three separate experiments. Significant differences are represented with * (*p* < 0.05), ** (*p* < 0.01), and *** (*p* < 0.001).

The porcine COCs were cultured for 44 h in IVM media containing 250 μM Sal, after cumulus cells were removed from the COCs, the *C-MOS*, *MEK*, *ERK1*, and *ERK2* mRNA expression levels in the oocytes were determined. We measured that the mRNA expressions in oocytes between different treatment groups were significantly higher than those in the control group (Figure 6C–F), and significantly increased the phosphorylated MAPK protein (Figure 6A,B). The mRNA expression of the oocyte maturation genes (*CDK1*, *Cyclin B*, and *GFP-1*) also increased significantly (Figure 6G).

### 3.6. The Addition of Sal Can Reduce the Apoptosis and Increase the Proliferation of PA Blastocysts

**Figure 7:**
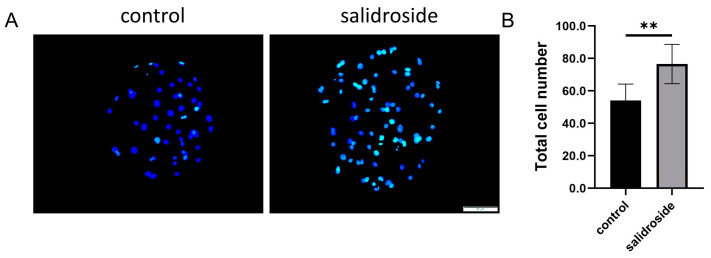
The addition of Sal can reduce the apoptosis of PA blastocysts and increase the proliferation of PA blastocysts. (**A**) Hoechst 33,342 staining of blastocysts on day 7 in the control and Sal groups. Scale bar = 50 μm. (**B**) Blastocyst total cell numbers in the control (n = 43) and Sal groups (n = 40). The data were obtained from three separate experiments. Significant differences are represented with ** (*p* < 0.01).

**Figure 8:**
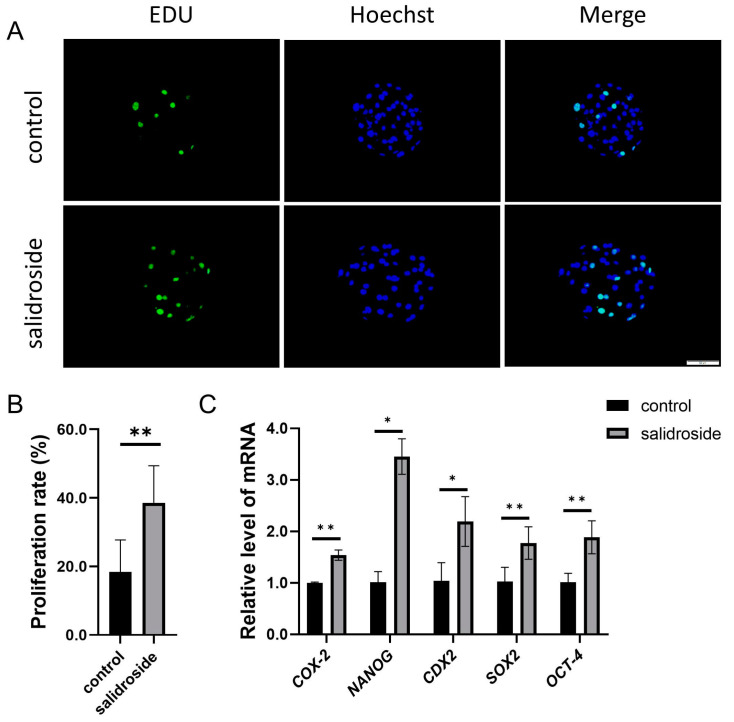
The addition of Sal can reduce the apoptosis of PA blastocysts and increase the proliferation of PA blastocysts. (**A**) Staining of EDU in blastocysts. Scale bar = 50 μm. (**B**) Rates of cell proliferation in blastocysts developing in the control (n = 61) and Sal groups (n = 67). (**C**) Relative gene expressions of *COX-2*, *NANOG*, *CDX2*, *SOX2*, and *OCT-4* at blastocyst stage in the control and Sal groups. The data were obtained from three separate experiments. Significant differences are represented with * (*p* < 0.05) and ** (*p* < 0.01).

We analyzed the total cell numbers and the ability of blastocysts to proliferate on day 6. The analysis of the total blastocyst cell number showed that it was significantly increased in the Sal-supplemented group compared with the untreated group (Figure 7). The number of blastocyst proliferations in the treatment group was significantly higher than that in the untreated group (Figure 8A,B), and Sal significantly increased the expression of *COX-2*, which is involved in blastocyst formation, and the expression of the pluripotency genes *NANOG*, *CDX2*, *SOX2*, and *OCT-4* (Figure 8C).

**Figure 9:**
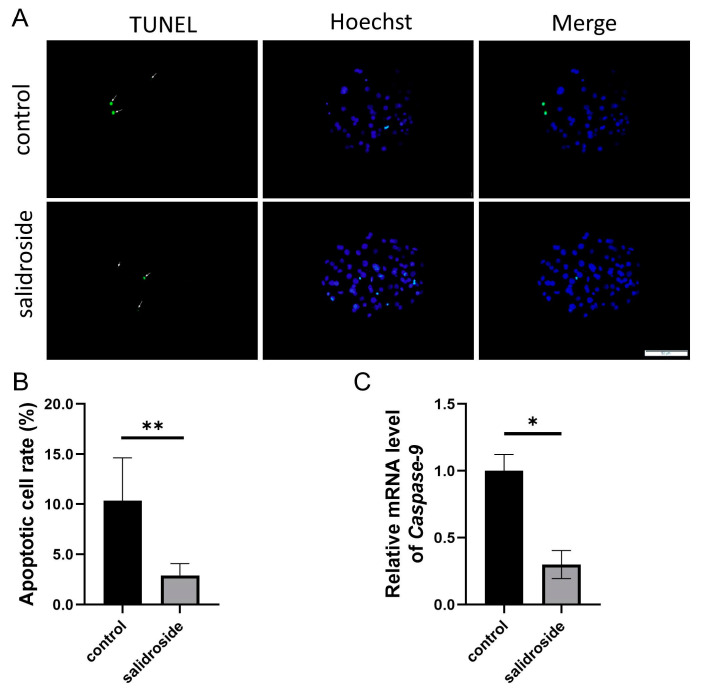
The addition of Sal can reduce the apoptosis of PA blastocysts and increase the proliferation of PA blastocysts. (**A**) Staining of TUNEL in blastocysts. Scale bar = 50 μm. (**B**) Rates of cell apoptosis in developing blastocysts in the control (n = 23) and Sal groups (n = 20). (**C**) Relative gene expressions of *Caspase-9* at the blastocyst stage in the control and Sal groups. The data were obtained from three separate experiments. Significant differences are represented with * (*p* < 0.05) and ** (*p* < 0.01).

Using TUNEL staining for the blastocysts obtained on the sixth day after parthenogenetic activation of oocytes in the treatment group and the untreated group, the number of apoptotic cells was significantly lower than that in the untreated group (Figure 9A,B), and down-regulated the apoptosis-related gene *Caspase-9* during the blastocyst stage (Figure 9C).

## 4. Discussion

Sal is a tyrosol-like glycoside that mainly exists in the roots of Rhodiola rosea plants, and has various biological and pharmacological properties. Numerous studies have been conducted over the past decade to investigate the medicinal properties of Sal, and anticancer, antioxidative, antiaging, antidiabetic, antidepressant, antihyperlipidemic, anti-inflammatory, and immunomodulatory effects were reported [19]. Although there have been studies indicating that Sal improves the lipid content in oocytes [20], we found that Sal promotes oocyte maturation while increasing mitochondrial activity, while reducing ROS and GSH levels in oocytes. This is reflected in aspects such as maturation, metabolism, cell communication, apoptosis, and oxidative stress. Our results provide new evidence for the improvement of oocyte quality and the subsequent effects of Sal.

To explore the impact of Sal on oocyte quality at a deeper level, considering the mechanism of oocytes and the in vivo physiology of oocyte maturation, the maturation of oocytes is closely related to the expansion of cumulus cells [21], cumulus cells are required for the transfer of energy or nutrients to support oocyte maturation. The highly active mitochondria in cumulus cells provide energy substrates for the maturation of oocytes [22], and have a critical role in protecting oocytes against oxidative stress [23]. The proliferative ability of cumulus cells is closely related to the nuclear and qualitative maturation of oocytes [24]. Sal promotes proliferation of vascular smooth muscle and PASMC by protecting mitochondria against oxidative stress [25,26]. Sal can reduce oxidative stress in human granulosa cell lines and reduce the occurrence of polycystic ovary syndrome [27]. Our results reveal the ability of Sal to promote the expansion of cumulus cells in terms of the energy supply to oocytes, which may be due to Sal reducing oxidative stress levels in cumulus cells and increasing mitochondrial DNA copy numbers.

Cumulus cell expansion is considered to be an important marker of oocyte maturation [28]. MPF consists of p-cdk1 and Cyclin B1, and is the main promoter of meiosis, which is essential for IVM in mammalian oocytes [29]. The activation of *MPF* and *ERK1/2* requires the activation of maternal *Ccnb1* and *MOS* mRNA translation [30], respectively. Taken together, the cooperation and positive feedback active action of *ERK1/2* and *CDK1* lead to the fine-tuning of mRNA translation and cell cycle progression during mouse oocyte maturation. MAPK plays a key role in promoting and stabilizing the spindle pole during oocyte meiosis [31], and the formation of the spindle structure is essential for chromosome segregation and cytokinesis [32]. Related studies have shown that in porcine oocytes, MAPK is mainly located at the poles of the metaphase spindle, and treatment with U0126 inhibits MAPK activity, resulting in the inhibition of chromosome segregation, PB1 discharge, and MII spindle formation [33]. The activation of MAPK plays an important role in oocyte maturation. Additionally, in most mammalian oocytes, GFP plays an important role in granulosa cell development and oocyte fertilization [34]. Sal can protect human bone marrow-derived endothelial progenitor cells [35], PC-12 cells [36], and alveolar epithelial cells through the activation of the ERK1/2 and MAPK signaling pathways [37]. The results indicate that Sal can increase the mRNA expression of the MAPK pathway (*C-MOS*, *MEK*, and *ERK1/2*) and that MAPK phosphorylation significantly increases, enhancing oocyte maturation by increasing the expressions of genes *(CDK1*, *CyclinB*, and *GFP-1*) related to oocyte maturation. This also suggests the potential mechanism by which Sal promotes oocyte maturation, which may be through activating the MAPK signaling pathway and maintaining spindle stability; further research is needed. This also explains the significantly higher exclusion rate of the first polar body in the treatment group.

Nevertheless, more studies are needed to further explore the mechanisms affecting maturity. There are many factors that affect the development of oocytes in vitro, among which oxidative stress is an important factor leading to oocyte damage [38]. Pig oocytes have a higher lipid content than other animals, and are particularly susceptible to oxidative stress [39], and GSH is an antioxidant that can scavenge ROS [40]. Particularly important in maintaining the redox state of the oocyte, a loss of GSH can lead to oxidative stress and the apoptosis of germ cells [41]. In related studies, Sal can increase the level of GSH and reduce the level of ROS in human umbilical vein endothelial cells (HUVECs) [42] and human RPE cells [43], and then reduce cell apoptosis to protect cells. In the present study, the addition of Sal during IVM reduced the accumulation of ROS in oocytes and increased the accumulation of GSH. We speculate that Sal may reduce oxidative stress by removing intracellular ROS and increasing GSH synthesis, thereby increasing the quality of porcine oocytes.

Mitochondria play an important role in the function of mammalian oocytes [44,45]. Mitochondria regulate the mitochondrial membrane potential through ATP [46]. Mitochondrial dysfunction and reduced mitochondrial membrane potential are the causes of poor oocyte quality and early embryo dysfunction [47]. That is, an abnormality of MMP is closely related to fertilization, germ cell development, and apoptosis [48]. Mitochondria synthesize adenosine triphosphate (ATP) via β-oxidation, a process that involves the electron transport chain [49]. During the meiotic maturation of oocytes, mitochondrial integrity is considered to be one of the indicators of cytoplasmic maturation [50]. Due to the complete maternal inheritance of mitochondria, mtDNA in oocytes is the only source of mtDNA in embryos [51], and numerous studies have shown a positive correlation between the mtDNA copy number and oocyte quality [18]. Numerous studies have shown that Sal can protect various cells, such as PC12 cells [52], HT22 cells [53], and 2BS cells [54], by regulating mitochondrial function and ROS levels. After treatment of diabetic mice with Sal, mitochondrial DNA copies and electron transfer chain proteins in the kidneys of mice significantly increased [55]. In our study, it was further confirmed that the MMP and mtDNA copy numbers of the treatment group significantly increased, and the ATP content significantly increased, which was also verified by previous experiments. The effect of Sal on promoting the maturation of oocytes may be due to the protection of mitochondria by Sal.

BCL2 family proteins, BCL2 and BAX, modulate mitochondrial membrane potential and activation of caspases [56]. In the mitochondrial apoptosis pathway, BCL2 has an anti-apoptotic role, whereas BAX promotes apoptosis following mitochondrial damage [57]. The protective effect of Sal on mitochondrial membrane potential and ATP production has been described above, and Sal inhibited cancer cell activation by inhibiting BCL2 in nasopharyngeal carcinoma cells [58] and human hepatocellular carcinoma [59]. Therefore, the expression levels of mitochondrial genes *BAX* and *BCL2* were analyzed. In our experiments we found via Q-PCR that Sal inhibited *BAX/BCL-2*. In further experiments, the expression of the *Caspase-3* gene and the expression of γH_2_AX protein were significantly reduced. This further illustrates that Sal protects against embryonic apoptosis and DNA damage based on mitochondria.

Oocyte quality is a key factor in determining embryo development [60]. Therefore, our study shows the treatment group after PA increases the developmental potential of the blastocysts. *COX2* affects the embryonic implantation ability during embryonic development [61]. In our results, SAL increased the expression of *COX-2* in blastocysts and the expression of totipotency-related genes (*OCT-4*, *CDX2*, *SOX2*, and *NANOG*) [62]. After EdU staining and TUNEL staining, the proliferation of PA blastocyst cells in the treatment group increased significantly, decreased apoptotic cells significantly, and the expression of the apoptosis gene *Caspase-9* slowed down the occurrence of apoptosis during embryonic development. This also further verified the positive effects of Sal on the quality and maturation of oocytes.

## 5. Conclusions

These results indicate that Sal modifies the expressions of key genes involved in oocyte cytoplasmic development and maturation, enhances their developmental ability, and is involved in modifying oocyte maturation, mitochondrial metabolism, apoptosis, and oxidative stress levels; these effects improve embryonic development and quality, and increase embryo yield, and support an optimized plan for efficient IVP embryo production.

## Figures and Tables

**Table 1:** Primer sequences of Mitochondrial DNA copy number in porcine oocyte for RT-PCR.

Gene Name	Sequence	Amplicon Size (bp)	Accession Number
*ATF6*	F: TATTTGCCTCTTTCATTGCCC R: GGATCGAGATTGTGCGGTTAT	123	NC_000845.1
*ND1*	F: GCCACATCCTCAATCTCCAT R: GATTAGAGGGTAGGGTATTGGTAG	99	NC_000845.1
*COX1*	F: TCCAATGGACATTATGGCTC R: GAAGACATCTCGGCTGAACT	220	NC_010443.5
*GCG*	F: GAATCAACACCATCGGTCAAAT R: CTCCACCCATAGAATGCCCAGT	198	NC_010457.5
*GADPH*	F: CCCAGAATATCATCCCTGCT R: CTGCTTCACCACCTTCTTGA	185	NC_010447.5

F, forward; R, reverse.

**Table 2:** The names of the tested genes, sequences of primers, sizes of PCR products, and accession numbers for RT-qPCR experiments.

Gene Name	Sequence	Amplicon Size (bp)	Accession Number
*Caspase3*	F: GTGGGATTGAGACGGACAGTGG R: TTCGCCAGGAATAGTAACCAGGTG	114	NM_214131.1
*BCL-2*	F: TGGATGACCGAGTACCTGAA R: CAGCCAGGAGAAATCAAACA	120	NM_001166486.1
*BA* *X*	F: CACCAAGAAGCTGAGCGAGTGT R: TCGGAAAAAGACCTCTCGGGGA	118	NM_173894
*COX2*	F: GGCTGCGGGAACATAATAGA R: GCAGCTCTGGGTCAAACTTC	183	NM_214321.1
*MEK*	F: TCATCGACTCCATGGCCAAC R: AGATGTCCGACTGCACGGAGTA	96	NM_001244550.1
*ERK1*	F: AGCCCTTTTGAGCATCAGACC R: AATGACGTTCTCGTGGCGG	84	XM_021088019.1
*ERK2*	F: CAAACCTTCCAACCTGCTGC R: TACTCCGTCAGGAACCCTGT	111	XM_021071922.1
*C-MOS*	F: GGTGGTGGCCTACAATCTCC R: TCAGCTTGTAGAGCGCGAAG	165	NM_001113219.1
*GDF9*	F: GTCTCCAACAAGAGAGAGATTC R: CTGCCAGAAGAGTCATGTTAC	109	NM_001001909.1
*CDK1*	F: TAATAAGCTGGGATCTACCACATC R: TGGCTACCACTTGACCTGTA	130	NM_001159304.2
*Cyclin B*	F: AGCTAGTGGTGGCTTCAAGG R: GCGCCATGACTTCCTGTA	101	NM_001170768.1
*NANOG*	F: AGGACAGCCCTGATTCTTCCACAA R: AAAGTTCTTGCATCTGCTGGAGGC	198	XM_021092390.1
*CDX2*	F: AGCCAAGTGAAAACCAGGAC R: TGCGGTTCTGAAACCAGATT	178	NM_001278769.1
*SOX2*	F: GCGGAGTGGAAACTTTTGTCC R: CGGGAAGCGTGTACTTATCCTT	157	NM_001123197.1
*OCT4*	F: GGCTTCAGACTTCGCCTCC R: AACCTGAGGTCCACAGTATGC	226	XM_021097869.1
*Caspase9*	F: GGCCACTGCCTCATCATCAA R: GGAGGTGGCTGGCCTTG	163	XM_013998997.2
*GADPH*	F: CCCAGAATATCATCCCTGCT R: CTGCTTCACCACCTTCTTGA	185	NM_001034034.2

F, forward; R, reverse.

## Data Availability

The data presented in this study are available on request from the corresponding author.

## Supplementary Materials

The following supporting information can be downloaded at: https://www.mdpi.com/article/10.3390/genes14091729/s1, Figure S1: Analysis of ROS levels and mitochondrial DNA copy number in cumulus cells.
