# Supplementary material for: Salidroside Supplementation Affects In Vitro Maturation and Preimplantation Embryonic Development by Promoting Meiotic Resumption
Source: Genes (Basel). 2023 Aug 30;14(9):1729. doi: 10.3390/genes14091729 (PMC10530922; doi:10.3390/genes14091729)
Supplement: Supplementary file 1 [file genes-14-01729-s001.zip › genes-2577472-supplementary.pdf]

Supplementary Data: Figure S1: Analysis of ROS levels and mitochondrial DNA copy number in cumulus cells.

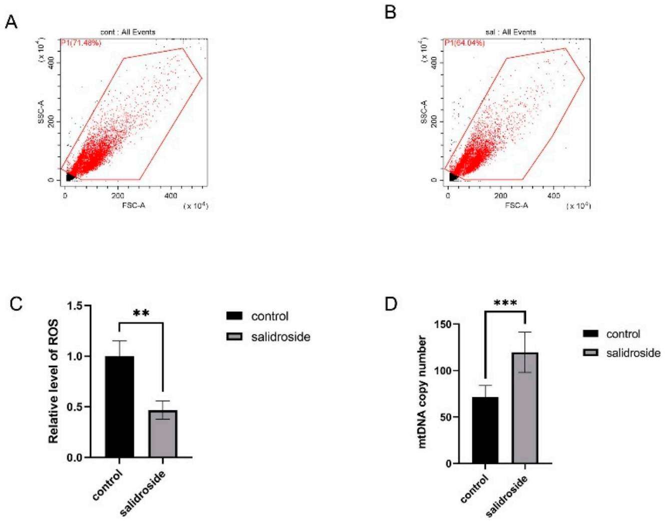

Figure S1. Analysis of ROS levels and mitochondrial DNA copy number in cumulus cells. (A) Flow cytometry scattergram of ROS level in cumulus cells in control group (B) Flow cytometry scattergram of ROS level in cumulus cells in treatment group (C) Relative level of ROS in cumulus cells in control group and treatment group (D) Mitochondrial DNA copy numbers of cumulus cells in control and treatment groups. The data were obtained from three separate experiments. Significant differences are represented with**($p < 0.01$) ***($p < 0.001$).
